# Associations between older African academics’ physical activity, walkability and mental health: a social distancing perspective

**DOI:** 10.1093/heapro/daab093

**Published:** 2021-09-20

**Authors:** Nestor Asiamah, Edgar Ramos Vieira, Kyriakos Kouveliotis, Janvier Gasana, Kofi Awuviry-Newton, Richard Eduafo

**Affiliations:** School of Health and Care Professions, University of Portsmouth, Winston Churchill Ave, Portsmouth PO1 2UP, UK; Department of Physical Therapy, Nicole Wertheim College of Nursing & Health Sciences, Florida International University, USA; Department of Health Management, Berlin School of Business and Innovation, Berlin, Germany; Faculty of Public Health, Department of Environmental and Occupational Health, Kuwait University, Kuwait; Faculty of Health and Medicine, Priority Research Centre for Generational Health and Ageing, University of Newcastle, Australia; Africa Centre for Epidemiology, Gerontology and Geriatric Care, Accra North, Ghana

**Keywords:** physical activity, neighborhood walkability, mental health, academics, social distancing, Africa

## Abstract

This study aimed to assess the moderating influence of neighborhood walkability on the association between physical activity (PA) and mental health among older African academics aged 50 years or more in cities with social distancing protocols in response to the Coronavirus disease 2019 (COVID-19). A total of 905 volunteer academics participated in the study. A hierarchical linear regression analysis was employed to conduct sensitivity analyses and test the study hypotheses. After controlling for sex, education and age, there was a positive association between PA and mental health. Neighborhood walkability moderated the relationship between PA and mental health, which suggests that during the pandemic PA was associated with higher mental health scores in more walkable neighborhoods. The study concludes that PA was beneficial to mental health in the social distancing context and was associated with higher mental health in more walkable neighborhoods, particularly in a social distancing context.

## INTRODUCTION

Research has shown that physical activity (PA) reduces the risk of non-communicable diseases (Steindorf *et al.*, 2012; Guure *et al.*, 2017), including cardiovascular conditions (e.g. stroke, diabetes, hypertension) and neurodegenerative disorders (e.g. Alzheimer’s disease, Parkinson’s disease) (Guure *et al.*, 2017; Asiamah *et al.*, 2020). The maintenance of PA over the life course also delays senescence (Rebelo-Marques *et al.*, 2018) and age-related morbidity and mortality (Reimers *et al.*, 2012; Asiamah *et al.*, 2020; Asiamah *et al.*, 2021). On the other hand, physical inactivity (PI) is a risk factor for the above diseases and is a leading cause of mortality (Asiamah, 2017; Rebelo-Marques *et al.*, 2018; Asiamah *et al.*, 2020; Asiamah *et al.*, 2021). Programs aimed at increasing PA and discouraging PI are, therefore, necessary to improve health at the individual, national and global levels. The promotion of PA as a health-seeking behavior is a typical example of these programs that plays a positive role toward the realization of the 2030 Sustainable Development Goal for health (Dai and Menhas, 2020). With this goal, stakeholders such as the World Health Organization expect to reduce by one-third premature mortality from non-communicable diseases through prevention and the promotion of mental health. This goal is in line with two health promotion models, namely the *Asset Model*, formulated by Morgan and Ziglio (Morgan and Ziglio, 2007), and the *salutogenesis theory*, developed by Antonovsky (Antonovsky, 1979). The Asset Model asserts that good health is maintained by utilizing protective factors or ‘health assets’ (e.g. walkable neighborhoods, social ties) in health-seeking behaviors (e.g. PA, exercise). This idea stems from the salutogenic view that good health can be maintained by making use of contextual resources (e.g. neighborhood services) to overcome stressors (e.g. poverty, aging) in the way of health-seeking behaviors such as PA.

Older adults are more susceptible to PI because aging is associated with factors that reduce PA (Gomes *et al.* 2017; Asiamah, 2017; Asiamah *et al.* 2020). These factors include physiological limitations, decline in social and financial resources and changes in life goals (Asiamah, 2017; Asiamah *et al.*, 2021). The willingness and ability to maintain PA and meet recommended levels reduce with age. PI is higher among those in occupations that encourage workaholism and involve prolonged sitting (George *et al.*, 2014; Hogan *et al.*, 2016). Workaholism is a compulsive professional behavior in which people work at the cost of their sleep and social functions (Hogan *et al.*, 2016). Workaholics including academics (George *et al.*, 2014; Hogan *et al.*, 2016) spend most of their time on work and, therefore, risk prolong occupational sitting. Academics have an average occupational sitting time of 8 h a day; the job tasks of university staff are largely sedentary and limit access to social and environmental resources that facilitate PA (Hogan *et al.*, 2016; Oyeyemi *et al.*, 2019). Academics are workaholics who constantly stick to their computers and jobs (Hogan *et al.*, 2016), and social distancing protocols in response to COVID-19 may have worsened PI in academics. Recently, Asiamah *et al.* (Asiamah *et al.*, 2021) found that individuals, including academics, reduced PA time to comply with national and institutional social distancing protocols. Rapanta *et al.* (Rapanta *et al.*, 2020) observed that an increase in online teaching reduced social engagement and PA in academics. Several studies reported that sedentary behavior and PI increased in the last 12 months as a consequence of social distancing measures and online teaching (Asiamah *et al.*, 2021; Cullen *et al.* 2020; Rapanta *et al.*, 2020). Therefore, PI is likely to increase faster in some occupations (e.g. teaching in a university) in a social distancing context.

Given COVID-19 social disruptions, many experts have alerted authorities to invest in the design of neighborhoods that support PA respecting social distancing guidelines (Dietz *et al.*, 2020; Megahed and Ghoneim, 2020; Nguyen *et al.*, 2020; Pinheiro and Luís, 2020). This effort has been influenced by studies that evidenced that walkable built environments can play a positive role in PA and social activity (Van Holle *et al.*, 2016; Barnett *et al.*, 2017; Edwards and Dulai 2018; Chen *et al.*, 2019). Neighborhood walkability is the degree to which a community provides resources (e.g. services, lorry parks, green spaces, road networks, destinations) that support PA (Barnette *et al.*, 2017; Chen *et al.*, 2019). PA can be maintained in walkable neighborhoods that allow for PA respecting social distancing guidelines (Pinheiro and Luís, 2020; Megahed and Ghoneim, 2020). For this reason, researchers have called for studies investigating the relationship between neighborhood walkability and PA, arguing that empirical research is needed to encourage stakeholders to implement the foregoing recommendations (Dietz *et al.*, 2020; Megahed and Ghoneim, 2020; Pinheiro and Luís, 2020).

Therefore, this study examined the moderating influence of neighborhood walkability in the association between PA and mental health among older African academics. Mental health was deemed the most appropriate outcome measure for this study because recent research has shown that mental health struggles are the most likely consequence of COVID-19 social distancing efforts (Asiamah *et al.*, 2021; Cullen *et al.*, 2020). Since mental health improvement is a key part of the 2030 SDG for health (Dai and Menhas, 2020), focusing on mental health in this study is an opportunity to demonstrate the joint role of neighborhood design and PA in the realization of the foregoing goal. With academics being among workers most vulnerable to PI, we deemed it necessary to provide an understanding of how PA can be supported by the built environment to buffer mental health declines in a social distancing context. This cross-sectional study sets the basis for potential prospective designs investigating whether neighborhood redesign, can reduce PI and its health risks associated with social distancing during a pandemic. Focusing on African academics in the current study is of significance because neighborhood walkability in Africa is among the lowest in the world (Asiamah, 2017; Oyeyemi *et al.*, 2019; Asiamah *et al.*, 2021), and neighborhood improvement interventions in the continent are rare (Asiamah, 2017; Asiamah *et al.*, 2021). Focusing on African academics living in less walkable neighborhoods gave us the opportunity to study a disadvantaged group facing a significant risk of PI. The two primary hypotheses tested were (i) PA is positively associated with mental health in older African academics, and (ii) the strength of the association between PA and mental health increases as neighborhood walkability increases.

## METHODS

### Design

This study adopted the cross-sectional design with a sensitivity analysis and measures against common methods bias.

### Participants and selection

The population of this study was full-time African academics aged 50 years or older who were observing national and institutional social distancing protocols while carrying out job tasks online or onsite. We operationally define ‘academics’ as academic staff involved in teaching and research in a tertiary institution. These staff may also be involved in administrative work in the university. We focused on universities in three African countries (Ghana, Kenya and Nigeria) that gave us access to a list of faculty members who were observing social distancing measures while working between August and October 2020. The lists received from 10 universities (Ghana = 3, Kenya = 3, Nigeria = 4) included the emails and other contact details of 2601 academics. We emailed all academics on the list to invite them to participate in the study. Over 8 weeks, we received replies from 1092 academics, out of which 922 agreed to participate. After waiting for an extra week, we received no new replies. We used the following inclusion criteria: (i) having at least a year of work experience as an academic; (ii) being aged 50 years or more and (iii) ability to read and write in English, the language in which questionnaires were administered. A total of 905 academics met these criteria. We gathered data on all eligible academics to maximize statistical power (George *et al.*, 2014; Asiamah *et al.*, 2021).

### Primary measures

The outcome variable is mental health, measured with a 9-item standard scale [Supplementary Appendix A (Asiamah *et al.*, 2021)]. This scale measured mental health status in the last 7 days using five descriptive anchors (i.e. *strongly disagree, disagree, somewhat agree, agree, strongly agree*) and produced Cronbach’s alpha coefficient = 0.82 in a similar African sample (Asiamah *et al.*, 2021). In the current study, it produced a Cronbach’s alpha coefficient = 0.94, which is more than the minimum coefficient of 0.7 recommended in the literature (Asiamah *et al.*, 2021).

Neighborhood walkability was measured with NEWS-A (the Australian version of the Neighborhood Environment Walkability Scale), an 11-item standard scale that measured the walkability of the neighborhood where the individual had lived in the past year and is associated with the same descriptive anchors as the mental health scale used. This scale (Supplementary Appendix B) was preferred to others because it is short, well suited for older adults and produced reliable findings in a recent study conducted in Africa (Asiamah *et al.*, 2021). Most of its items are indicators of neighborhood sociability, which is the core feature of walkable communities (Oyeyemi *et al.*, 2014). It is, therefore, more suited for studies measuring activities (e.g. PA) undertaken as part of socialization in the community. Other scales are longer and had high non-response rate in similar populations (Oyeyemi *et al.*, 2014; Asiamah *et al.*, 2021). This questionnaire produced a Cronbach alpha value of 0.8 in the current study and a value of 0.89 in a previous study on a similar sample (Asiamah *et al.*, 2021).

PA was assessed using the short form of the International Physical Activity Questionnaire (SF-IPAQ) (see Supplementary Appendix C). This measure is validated (Oyeyemi *et al.*, 2014; Lavelle *et al.*, 2020); it includes three domains of PA (i.e. vigorous PA, moderate PA and walking), and has satisfactory reliability and validity scores on African samples (Oyeyemi *et al.*, 2014). It measured PA performed by the individual in the last 7 days. An index from the scale was computed with the standard formula (Lavelle *et al.*, 2020):

Total MET-minutes/week=Vigorous PA (MET*minutes*days)+Moderate  PA (MET*minutes*days)+Walking (MET*minutes*days)

MET in the formula stands for Metabolic Equivalent whereas vigorous PA, moderate PA and walking are the three dimensions of PA. The MET levels assigned to these dimensions are walking = 3.3; moderate PA = 4 and vigorous PA = 8.

#### Identification and measurement of confounding variables

The disengagement theory of aging (DTA) argues that PA is a function of age and socio-economic status (SES) variables (e.g. education, income and employment) (Cumming *et al.*, 1960). A recent review of key aging theories also suggests that household or individual income, sex, education and age are the primary determinants of PA (Asiamah, 2017). Thus, this review and the DTA imply that SES, sex and age can affect PA and its relationship with mental health and related outcome variables. Since job tenure and chronic disease status (CDS) (i.e. whether the individual had one or more clinically diagnosed conditions or not) are personal variables that change with time (i.e. age), they can also affect one’s PA. Hogan *et al.* (Hogan *et al.*, 2016) have attributed low PA level to workaholism caused by multiple job roles generally held by academics. If so, having more than one job role (i.e. measured as ‘alternative role(s)’) can influence PA. Finally, university campuses may offer limited access to neighborhood services and resources for PA; hence, academics living on and off-campus may have different contextual support for PA. As such, age, income, education, sex, CDS, job tenure, campus residency and alternative role(s) can confound the study hypotheses. Age, income, education and job tenure were measured as continuous variables. Specifically, education was measured as the total number of years of schooling, job tenure as the number of years the individual had worked as an academic, and income as gross monthly income in United States Dollars. Sex (male—0, female—1), campus residency (No—0; Yes—1), alternative roles (No—0; Yes—1) and CDS (None—0; one or more—1) were measured as nominal variables that were dummy-coded to support regression analysis.

### The questionnaire and steps against common methods bias

A questionnaire integrating all measures was used to gather data. The survey comprised four sections. The first section was an introductory statement including the purpose of the study, eligibility criteria and survey completion instructions. The second section included five screening questions and measures of PA. The third section included the demographic characteristics and confounding variables. The fourth section presented measures on neighborhood walkability and mental health. Our arrangement of the parts and items was based on Joran and Troth’s (Joran and Troth, 2020) recommendations for avoiding common methods bias. The introductory section of the questionnaire explained the benefits of the study to academics, encouraging participants to provide honest and objective responses. At the data analysis stage, we adopted *Harman’s one-factor test*, the commonest statistical assessment of common methods bias, to further evaluate our data (Chang *et al.*, 2010; Jordan and Troth, 2020). The mental health and neighborhood walkability scales produced 3 and 4-factor solutions, respectively, with items of the scales producing factor loadings ≥0.5. These results satisfy rules of thumb established in the literature for Harman’s one-factor test and, therefore, indicated that common methods bias was avoided or sufficiently minimized (Chang *et al.*, 2010; Jordan and Troth, 2020).

### Ethics and data collection

This study was approved by an institutional ethics review board (No. 03-ACE2020). All participants agreed to participate in the study voluntarily and signed an informed consent form delivered at the time of participant selection. The survey, which was designed with Google Forms to avoid more than one response from the same participant, was sent to participants via email as a hyperlink. The email asked participants to follow the link to a pop-up questionnaire that could be completed with a relatively weak network. To submit the questionnaire, respondents had to click an icon following the final question on the questionnaire, after which instant feedback was sent to the researchers with the relevant tracking code. Respondents did not have to download the questionnaire. With the help of our information technology consultants, we assigned unique codes to each email and response feedback to track potential extra responses from other devices of the same participant.

We piloted the questionnaire and the above measures in two ways. First, three researchers who had used a similar data collection procedure were asked to review the online questionnaire to establish face validity. We subsequently piloted the survey on 50 participants randomly selected from our sample. At this stage, we asked the participants to identify and report survey ambiguities and errors, and any challenges they faced in completing the questionnaire. Responses from 39 participants showed that the survey was without issues. We confirmed the usability of the pilot survey with satisfactory Cronbach’s alpha coefficients on key constructs (Mental health = 0.92; neighborhood walkability = 0.89) (Srinivasan and Lohith, 2017). Questionnaires were administered and completed over 4 weeks (15 November to 16 December 2020). A total of 766 questionnaires were completed after one or two follow-up phone calls to participants who did not respond within the first 2 weeks. After applying the eligibility criteria and 5 screening questions, 67 questionnaires were dropped. After further removing 6 duplicate responses, we analyzed data from 693 questionnaires.

### Statistical analysis method

Data analysis was conducted with SPSS 25 for Windows. The exploratory phase of the analysis was focused on identifying missing data and outliers and knowing whether the data would support a parametric test. Six (6) questionnaires that contained missing data were not removed as their missing data were <10% and were randomly distributed (Madley-Dowd *et al.*, 2019). Data distribution was assessed with Shapiro−Wilk’s test as well as descriptive statistics, namely mean, standard deviation, skewness and kurtosis. This analysis evidenced a satisfactory distribution of the data (i.e. skewness = 0.06; kurtosis = 1.21, Shapiro−Wilk’s statistic = 0.188, *p* = 0.108) associated with the dependent variable—mental health. We then employed a sensitivity analysis adopted from previous studies to screen for confounding variables, ensuring that only variables likely to confound the primary hypotheses were incorporated into the final analysis (Rothman and Greenland, 1998; Asiamah *et al.*, 2021). In this vein, we fitted univariate regression models to estimate crude standardized regression coefficients (β) representing the influence of PA on mental health, and the influences of the potential confounding variables on PA. We removed confounding variables with *p* < 0.25. Subsequently, a multiple regression model was used to assess the influences (i.e. β coefficients) of PA and the confounding variables on mental health. Confounding variables that led to a 10% change in the crude regression coefficient between PA and mental health were considered ultimate confounders.

The hypotheses were tested with hierarchical linear regression analysis. The first hypothesis was tested with two regression models alongside a second sensitivity analysis. The first baseline model, tested the relationship between PA and mental health without adjusting for the ultimate confounding variables. The second adjusted for the ultimate confounding variables. The conclusions of the study are based on this adjusted model. We compared the coefficients and explained variances between the first baseline and ultimate models to demonstrate the significance of the confounding variables. To test the second hypothesis, we first computed a dummy variable, which is an interaction term between PA and neighborhood walkability (i.e. PA*NW). We fitted two extra regression models (i.e. the second baseline and ultimate models) to assess the relationship between the interaction term and mental health. With this procedure, we aimed to assess *pure moderation* (Asiamah *et al.*, 2021) to understand whether neighborhood walkability significantly increased the regression coefficient between PA and mental health. Pearson’s correlation coefficients for relevant pairs of variables were computed before testing the hypotheses. The statistical significance of the results was detected at *p* < 0.05. Relevant other assumptions governing the use of multiple linear regression analysis (e.g. multi-collinearity and independence of errors) were assessed and met for all regression models fitted.

## FINDINGS

As Table 1 indicates, 27% (*n* = 189) of older academics were from Ghana, 48% (*n* = 334) were from Nigeria and 25% (*n* = 170) were from Kenya. About 31% (*n* = 218) of academics were women whereas 68% (*n* = 475) were men. The average PA was about 4750 MET-minutes/week (Mean = 4750; SD = 2716) and the average mental health score was about 32 (Mean = 32; SD = 8). The average level of neighborhood walkability was 28 (Mean = 27.8; SD = 6.6). The average age of participants was 56 years (Mean = 56.26; SD = 5.36). Table 2 shows results of the sensitivity analysis.

**Table 1:** Primary participant’s characteristics (*n* = 693)

Variable	Level	Frequency^a^/Mean^b^	Percent (%)^a^/SD^b^
Country	Ghana	189	27.3
	Nigeria	334	48.2
	Kenya	170	24.5
	Total	693	100
Gender	Male	475	68.5
	Female	218	31.5
	Total	693	100
Residency	No	543	78.4
	Yes	150	21.6
	Total	693	100
Alternative role(s)	No	429	61.9
	Yes	264	38.1
	Total	693	100
Chronic disease status	None	407	58.7
	≥1	286	41.3
	Total	693	100
Income (USD)	−	1121.01	302.33
Tenure (years)	−	15.08	7.03
Education (years)	−	12.66	3.21
Age (years)	−	56.26	5.36
Mental health	−	32.23	7.71
PA (MET-minutes/week)	−	4750.36	2716.46
Neighborhood walkability	−	27.79	6.22

a Applies to categorical variables.

b Applies to continuous variables

−, not applicable; USD, United States Dollars; PA, physical activity; SD, standard deviation; MET, metabolic equivalent.

**Table 2:** Variables removed and retained in the sensitivity analysis (*n *=* *693)

Model	Primary predictor	Stage 1			Stage 2		
		β	*t*	*p*	Adjusted β	Change in β	% Change in β
1^c^	PA (MET-minutes/week)	0.256	6.97	0.000	−	−	−
2^d^	Gender (reference—male)	−0.204	−5.563	0.000	0.285	0.029	11%
	Education	−0.087	−2.177	0.030	0.223	−0.033	−13%
	Income (USD)^b^	−0.249	−6.781	0.000	0.265	0.009	4%
	Residency (reference—No)	0.088	2.309	0.021	0.284	0.028	11%
	Tenure^b^	0.185	4.148	0.000	0.262	0.006	2%
	Alternative role(s)^b^ (reference—No)	0.059	1.592	0.112	0.25	−0.006	−2%
	CDS^a^ (reference—None)	−0.032	−0.823	0.411	−	−	−
	Age (years)	−0.218	−5.636	0.000	0.329	0.073	29%

a Variables removed in stage 1.

b Variables removed in stage 2.

c Model assessing the relationship between PA and mental health.

d Model assessing the relationship between potential confounders and PA.

−, not applicable; PA, physical activity; USD, United States Dollars; CDS, Chronic Disease Status; MET, metabolic equivalent;

In Table 2, the crude regression coefficient between PA and mental health was 0.26 (β = 0.256, *t* = 6.97, *p* < 0.001). In the first stage, only CDS had a *p* > 0.25 and was removed from the analysis. In the second stage, income, tenure and alternative role(s) were removed from the analysis as they accounted for <10% of a change in the crude coefficient between PA and mental health. Thus, sex, education, residency and age were incorporated into the final analysis as the ultimate confounding variables. Table 3 presents Pearson’s correlation between relevant variables.

**Table 3:** The correlation between neighborhood walkability, physical activity and mental health among older academics (*n* = 693)

Variable	1	2	3	4	5	6	7	8
1. Mental health	1	0.256^**^	0.259^**^	0.315^**^	0.063	−0.208^**^	−0.143^**^	0.236^**^
2. PA (MET-minutes/week)		1	0.075^*^	0.911^**^	−0.227^**^	−0.156^**^	0.148^**^	−0.232^**^
3. Neighborhood walkability			1	0.429^**^	−0.037	−0.141^**^	0.255^**^	−0.035
4. PA^*^NW				1	−0.228^**^	−0.155^**^	0.225^**^	−0.204^**^
5. Gender (Reference—male)					1	0.087^*^	−0.054	0.015
6. Education (years)						1	0.077^*^	0.239^**^
7. Residency (reference—No)							1	−0.131^**^
8. Age (years)								1

* *p* < 0.05.

** *p* < 0.001.

−, not applicable; SD, standard deviation; PA, physical activity; NW, neighborhood walkability; MET, metabolic equivalent.

In Table 3, mental health was positively correlated with PA (*r* = 0.26, *p* < 0.001, two-tailed) and neighborhood walkability (*r* = 0.26, *p* < 0.001, two-tailed). This result indicates that mental health increased as PA and neighborhood walkability increased. The interaction term (i.e. PA*NW) is also positively correlated with mental health (*r* = 0.32, *p* < 0.001, two-tailed). All the confounding variables were significantly correlated with PA at *p* < 0.001, which implies that the primary relationships could be confounded by the ultimate covariates and reinforces the importance of the sensitivity analysis.

As presented in Table 4, PA was positively associated with mental health in the baseline model (β = 0.26, *t* = 6.97, *p* < 0.001). In the first ultimate model (i.e. Model 2 in Table 4), PA was more strongly correlated with mental health (β = 0.35, *t* = 9.68, *p* < 0.001). Models 3 and 4 assess the second hypothesis. With Model 3, the interaction term is positively associated with mental health (β = 0.32, *t* = 8.71, *p* < 0.001), but this relationship is stronger in the fourth model (β = 0.42, *t* = 11.98, *p* < 0.001). Thus, the ultimate coefficient between PA and mental health (β = 0.35) increased by ∼17% in the fourth model (β = 0.42) due to neighborhood walkability.

**Table 4:** The association between neighborhood walkability, physical activity and mental health among older academics (*n* = 693)

Model	Predictor	Coefficients			95% CI	Tolerance	Model fit					
		*B*	SE	β(*t*)			*R* ^2^	Adjusted *R*^2^	Change in *R*^2^	Durbin-Watson	*F*	*p*
1	(Constant)	28.77	0.57	(50.36)^**^	±2.24	−	0.07	0.06	0.01	−	48.58	0.000
	PA (MET-minutes/week)	0.00	0.00	0.26 (6.97)^**^	±0.00	−						
2	(Constant)	11.98	3.31	(3.62)^**^	±13.00	−	0.25	0.24	0.01	1.69	43.93	0.000
	PA (MET-minutes/week)	0.00	0.00	0.35 (9.68)^**^	±0.00	0.88						
	Gender (ref.—male)	2.56	0.58	0.15 (4.46)^**^	±2.26	0.95						
	Education (years)	−3.77	0.54	−0.24 (−6.93)^**^	±2.14	0.91						
	Residency (ref.—No)	−2.24	0.65	−0.12 (−3.46)^*^	±2.54	0.95						
	Age (years)	0.52	0.05	0.35 (9.94)^**^	±0.20	0.89						
3	(Constant)	28.44	0.52	(55.01)^**^	±2.03	−	0.099	0.098	0.001	−	75.88	0.000
	PA^*^NW	0.29	0.03	0.32 (8.71)^**^	±0.13	−						
4	(Constant)	11.40	3.15	(3.62)^**^	±12.38	−	0.294	0.289	0.005	1.73	55.53	0.000
	PA^*^NW	0.39	0.03	0.42 (11.98)^**^	±0.13	0.86						
	Gender (ref.—male)	2.76	0.56	0.17 (4.96)^**^	±2.19	0.95						
	Education (years)	−3.53	0.53	−0.23 (−6.68)^**^	±2.08	0.91						
	Residency (ref.—no)	−3.12	0.64	−0.17 (−4.90)^**^	±2.50	0.92						
	Age (years)	0.51	0.05	0.35 (10.14)^**^	±0.20	0.90						

* *p* < 0.05.

** *p* < 0.00.

−, not applicable; SE, standard error; CI, confidence interval; PA, physical activity; NW, neighborhood walkability; MET, metabolic equivalent.

**Fig. 1:**
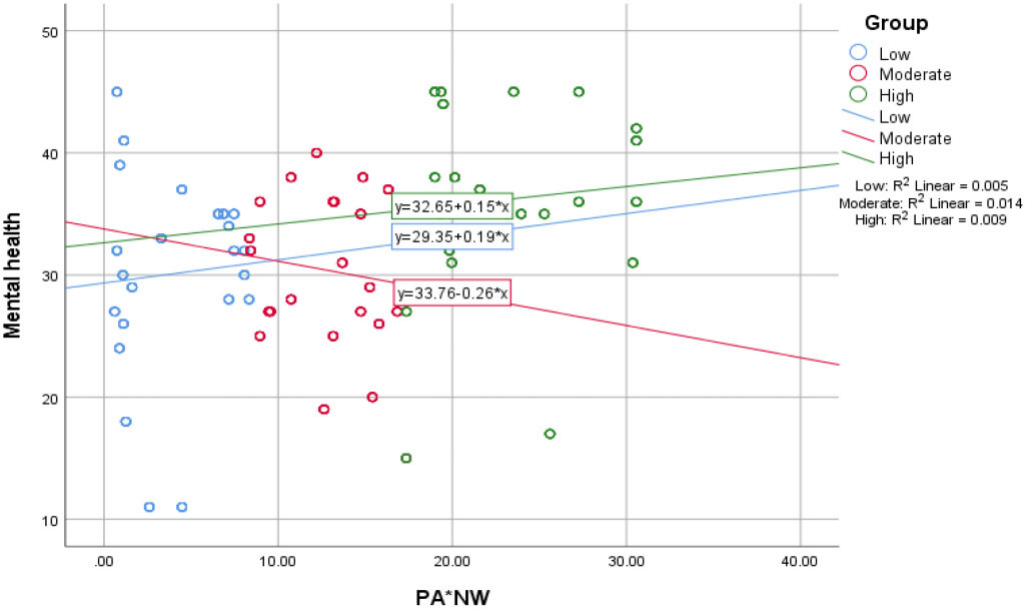
The relationship between mental health and different levels of the interaction between neighborhood walkability and physical activity (*n* = 693; low = 231; moderate = 231; high = 231).

Figure 1 depicts the strength of the relationship between mental health and the interaction term, which was dummy-coded into three groups (i.e. low, moderate and high). The variance accounted by the regression model increased between low and moderate as well as between low and high, which supports the confirmation of the second hypothesis.

## DISCUSSION

This study assessed the relationship between PA and mental health as well as the moderating influence of neighborhood walkability on this relationship among older African academics aged 50 years or more affected by social distancing rules due to COVID-19. There was a positive association between PA and mental health after controlling for confounding variables. This result suggests that the mental health of older academics in a social distancing context increases with PA. It also supports the activity theory of aging, which assumes that the maintenance of PA over the life course is beneficial to health (Havighurst, 1961). The gerontological literature recognizes mental health as a key indicator of health in older and aging people (Callow *et al.*, 2020; Wermelinger *et al.*, 2018). Our findings suggest that PA can be associated with higher mental health and its related conditions such as quality of life. Our result is also consistent with a growing body of studies that have investigated the relationship between PA and mental health in older adults (Lautenschlager *et al.*, 2004; Kadariya *et al.*, 2019; Callow *et al.*, 2020). In North America, Callow *et al.* (Callow *et al.*, 2020) confirmed a positive relationship between PA and mental health among 1046 older adults observing social distancing measures. In a PA intervention in the UK, Fox *et al.* (Fox *et al.*, 2007) confirmed that PA is positively associated with mental health among older adults. A scoping review by Kadariya *et al.* (Kadariya *et al.*, 2019) showed that mental health improves as PA increases among older adults in South-Eastern Asia. While these pieces of evidence are supported by our result, the current study is unique because it focused on older African academics in a social distancing context due to COVID-19. The key lesson learned is that PA can be beneficial in contexts where efforts toward containment of a pandemic necessitate social distancing, restrict social interactions and limit access to neighborhood resources.

The study also found a positive association between the interaction term between PA and neighborhood walkability (i.e. PA*NW) and mental health. PA was likely to more strongly predict mental health in more walkable neighborhoods that are characterized by green spaces, parks, essential services, cross-walks, pavements, and spacious roads and streets (Van Holle *et al.*, 2016; Van Cauwenberg *et al.*, 2016). By confirming the second hypothesis, this study supports traditional person−environment (P−E) fit paradigms such as Lawton’s (Lawton, 1989) P−E fit model and Cantor’s (Cantor, 1975) framework. These models suggest that behaviors such as physical and social activities are a function of the individual and the environment. Thus, people’s PA is influenced by resources available in their neighborhoods as well as their individual conditions (e.g. functional capacity, income and social support). Wahl and Gerstorf (Wahl and Gerstorf, 2018) developed the Context Dynamics in Ageing (CODA) framework to advance the imports of Lawton’s (Lawton, 1989) and Cantor’s (Cantor, 1975) P−E fit models. The CODA better aligns with our result as it describes how walkability factors such as services, parks and green spaces can moderate behaviors (e.g. social activity, PA) to improve health. The congruence of the above result with these P−E fit models adds to the contextual uniqueness of this study.

Our findings have several implications for gerontology, health promotion and occupational health. Interventions focusing on the improvement of neighborhood walkability and the walkability of university campuses are necessary, especially in African countries where national PA programs are unavailable (Oyeyemi *et al.*, 2014; Asiamah, 2017; Asiamah *et al.*, 2021a,b,c). While universities may adopt a policy emphasizing continuous improvement and investment in campus walkability and PA (sporting) facilities, governments ought to invest in neighborhood green spaces, essential services, parks, and accessible road and commercial infrastructure, which constitute the underlying attributes of highly walkable neighborhoods (Wahl and Gerstorf, 2018; Asiamah *et al.*, 2021). These interventions must accompany a change in academics’ attitude toward PA and reduction of occupational sitting, to meet recommended PA levels. These steps by academics, universities and governments are necessary responses to occupational health promotion efforts in gerontology that have received little attention in African and other developing countries (Oyeyemi *et al.*, 2014; Asiamah, 2017; Asiamah *et al.*, 2021).

From an occupational health perspective, universities and education ministries have to value and prioritize employee health as an approach to maximizing productivity, reduce illness and improve quality of life. Needless to say, workforces are less productive without optimal health because work engagement can be associated with physical, emotional and psychological wellbeing. Given future pandemics and epidemics and that COVID-19 may take a long time to eradicate (Asiamah *et al.*, 2021; Cullen *et al.*, 2020), universities need to adopt occupational health programs that provide academics access to sporting facilities and PA-oriented services and campus resources. Though these interventions may require a relatively large expenditure, they would contribute to manpower and financial productivity in the long-term by reducing turnover associated with sick leaves, hospitalization, early retirement and death especially among older staff. Nevertheless, the implementation of the above recommendations should be based on additional future and longitudinal research that enhances an understanding of how much neighborhood walkability projects or interventions can encourage PA and increase mental health over time.

Since this study adopted a cross-sectional design, future prospective designs investigating the effect of PA and the interaction term (i.e. PA*NW) on mental health are necessary. Randomized longitudinal designs can be effective at evidencing the long-term effects of PA and the interaction term on mental health or any other health outcome. Given a possible sampling bias accompanied by our use of non-probability sampling and a non-powered sample, future studies employing representative samples are needed to guarantee the generalizability of our evidence. Despite the above limitations, our study adds to the gerontology literature by evidencing the potential contribution of the neighborhood and PA to health in a social distancing context. Moreover, our sensitivity analysis and steps against common methods bias have not only enhanced the internal validity of the findings but can serve as a model for future research, given that most cross-sectional studies did not adjust for confounding variables or used the wrong methods to address confounding (DeMaris, 2014; Asiamah *et al.*, 2019). The importance of our methodology is bolstered by the necessity of sensitivity analyses and efforts against common method bias as captured in the STROBE (Strengthening the Reporting of Observational Studies in Epidemiology) (Von Elm *et al.*, 2014).

Additionally, our sensitivity analysis for confounding has implications and lessons for future research. Without adjusting for the confounding variables, the study would have reported a lower association between PA and mental health. The strength of the relationship between the interaction term and mental health would also have been under-estimated. An increase in the regression coefficients in the two ultimate models is consistent with the argument of Asiamah *et al.* (Asiamah *et al.*, 2019) that confounding can lead to under- or over-estimation of the ultimate effect coefficients. Originators of the sensitivity analysis employed in this study (Rothman and Greenland, 1998) also reasoned that failing to adjust for confounders can result in an under- or over-estimated effect size. That is, adjustment for confounding variables does not always result in smaller ultimate coefficients (vis à vis the crude coefficients) and can result in effect sizes that are larger than the crude coefficients.

## CONCLUSION

An increase in PA is associated with higher mental health scores taking into account confounding personal factors. Higher mental health scores in older African academics were associated with PA in a COVID-19 social distancing situation. The relationship between PA and mental health was stronger in more walkable neighborhoods. Hence, interventions focusing on the provision of walkable neighborhoods could increase PA and mental health in a pandemic context where social distancing measures may limit access to neighborhood resources.

## ETHICAL APPROVAL

This study received ethical approval from Africa Centre for Epidemiology’s Ethics Review Board (No. 03-ACE2020). All participants consented to participate in the study voluntarily.

## SUPPLEMENTARY MATERIAL

Supplementary material is available at *Health Promotion International* online.

## AUTHORS’ CONTRIBUTIONS

N.A. conceived the research idea, analyzed the data and wrote the original manuscript. E.R.V., K.K., J.G., K.A. and R.E. contributed to the manuscript. All authors proofread and approved the manuscript.

## Supplementary Material

daab093_Supplementary_Data
