# Supplementary material for: Associations between older African academics’ physical activity, walkability and mental health: a social distancing perspective
Source: Health Promot Int. 2021 Sep 20;37(2):daab093. doi: 10.1093/heapro/daab093 (PMC8500010; doi:10.1093/heapro/daab093)
Supplement: daab093_Supplementary_Data [file daab093_supplementary_data.zip › daab093-suppl_data/Appendix A. Mental health scale.doc]

Appendix A. Items of the mental health scale

| Variable | 1 | 2 | 3 | 4 | 5 |
| --- | --- | --- | --- | --- | --- |
| I am often carefree and in good spirits. |  |  |  |  |  |
| I enjoy my life. |  |  |  |  |  |
| All in all, I am satisfied with my life. |  |  |  |  |  |
| In general, I am confident |  |  |  |  |  |
| I manage well to fulfil my needs |  |  |  |  |  |
| I am in good physical and emotional condition |  |  |  |  |  |
| I feel that I am well equipped to deal with life and its difficulties |  |  |  |  |  |
| Much of what I do brings me joy |  |  |  |  |  |
| I am a calm, balanced human being |  |  |  |  |  |
| Mental health |  |  |  |  |  |

¶This table shows items of the scale used to measure mental health. Descriptive anchors: 1 = strongly disagree; 2 = disagree; 3 = somewhat agree; 4 = agree, and 5 = strongly agree.
