# Supplementary material for: Associations between older African academics’ physical activity, walkability and mental health: a social distancing perspective
Source: Health Promot Int. 2021 Sep 20;37(2):daab093. doi: 10.1093/heapro/daab093 (PMC8500010; doi:10.1093/heapro/daab093)
Supplement: daab093_Supplementary_Data [file daab093_supplementary_data.zip › daab093-suppl_data/Appendix B. Neighborhood walkability scale.doc]

Appendix B. Items of Neighborhood Sociability

|  | Item | 1 | 2 | 3 | 4 | 5 |
| --- | --- | --- | --- | --- | --- | --- |
| 1 | Many places are easy to go within walking distance |  |  |  |  |  |
| 2 | It is easy to walk to a public transport stop |  |  |  |  |  |
| 3 | There are footpaths along most of the streets |  |  |  |  |  |
| 4 | There are crosswalks and pedestrian signals |  |  |  |  |  |
| 5 | The streets in my neighbourhood are not hilly |  |  |  |  |  |
| 6 | Walkers in my neighbourhood can easily be seen |  |  |  |  |  |
| 7 | There is lots of greenery around my neighbourhood |  |  |  |  |  |
| 8 | There are many interesting things to look at |  |  |  |  |  |
| 9 | There is not much traffic along nearby streets |  |  |  |  |  |
| 10 | My neighbourhood has parks and walking trails |  |  |  |  |  |
| 11 | Crime rate in my neighbourhood is not a problem |  |  |  |  |  |

¶This table shows items of the Australian version of the Neighbourhood Environment Walkability Scale (NEWS-A). Descriptive anchors: 1 = strongly disagree; 2 = disagree; 3 = somewhat agree; 4 = agree, and 5 = strongly agree.
