# Supplementary material for: Associations between older African academics’ physical activity, walkability and mental health: a social distancing perspective
Source: Health Promot Int. 2021 Sep 20;37(2):daab093. doi: 10.1093/heapro/daab093 (PMC8500010; doi:10.1093/heapro/daab093)
Supplement: daab093_Supplementary_Data [file daab093_supplementary_data.zip › daab093-suppl_data/APPENDIX C.doc]

**APPENDIX C: SF-IPAQ (Abridged Version)**

Physical activity is any activity that increases your heart rate and makes you get out of breath some of the time e.g., jogging, brisk/fast walking, sports, and farming.

1. During the past 7 days, on how many days did you do **vigorous physical activities** (e.g., jogging, carrying heavy weights) for at least 10 minutes per day? ……….. Days
2. How much time did you spend doing **vigorous activities** on one of those days?

……… hours	……. Minutes

3. During the past 7 days, on how many days did you do **moderate physical activities** (e.g., Cleaning at home, walking quickly, bicycling) for at least 10 minutes per day? ……….. Days
4. How much time did you spend doing **moderate activities** on one of those days?

……… hours	……. Minutes

5. During the last 7 days, on how many days did you walk for at least 10 minutes at a time? ……………. Days per week
6. How much time did you spend walking on one of those days?

……… hours	……. Minutes

7. In the last one week, how much time did you spend seated idle or with screens (e.g., computers, phones, TV etc.) per day? ……… hours	……. Minutes

END OF QUESTIONNAIRE
